# The immediate pain relief of low-level laser therapy for burning mouth syndrome: a retrospective study of 94 cases

**DOI:** 10.3389/froh.2024.1458329

**Published:** 2024-12-18

**Authors:** Wenxin Mu, Shanshan Li, Qian Lu, Juan Wang, Xiaoan Tao

**Affiliations:** ^1^ Hospital of Stomatology, Sun Yat-sen University, Guangzhou, China; ^2^ Guangdong Provincial Clinical Research Center of Oral Diseases, Sun Yat-sen University, Guangzhou, China; ^3^ Guanghua School of Stomatology, Sun Yat-sen University, Guangzhou, China

**Keywords:** low-level laser therapy, burning mouth syndrome, pain management, visual analogue scale, retrospective study

## Abstract

**Significance:**

Burning mouth syndrome (BMS) is a chronic orofacial pain disorder that seriously affects quality of life of patients. In recent years, Low-level laser therapy (LLLT) has been regarded as an important innovation in pain management, but there is insufficient evidence of its effectiveness in patients with painful BMS. This study aimed to evaluate the efficacy of LLLT for immediate pain relief due to BMS.

**Methods:**

This retrospective study included 94 BMS patients that were treated with an intraoral semiconductor laser (635 nm, 100 mW/cm^2^). Pain was self-assessed before and immediately after LLLT sessions using a 0-to-10 visual analogue scale (VAS). Paired-samples *t*-test and multivariable binary logistic regression were used to analyze overall efficacy and its influencing factors.

**Results:**

After standardized LLLT session, 71.3% of patients reported an immediate pain decrease. Compared to pre-LLLT treatment, the VAS immediately post-LLLT was significantly reduced (*P* < 0.001). Mean post-LLLT VAS reduction was 2.2 ± 2.0, equivalent to 39.9% of the initial pain level. Meanwhile, low VAS before treatment, history of smoking or alcohol, xerostomia, and gingival lesions correlated with worse LLLT efficacy. There were no side effects or adverse reactions were noticed by the practitioner or reported by the patients.

**Conclusions:**

LLLT may provide non-pharmacological, non-invasive, side-effect-free, and rapid pain relief for painful BMS patients. No baseline characteristics affecting overall efficiency were found except for VAS before treatment, history of smoking or alcohol, xerostomia, and gingival lesions.

## 1 Introduction

Burning mouth syndrome (BMS) is a chronic orofacial pain disorder mainly found in middle-aged or elderly women, with a prevalence ranging from 0.01% to 3.9% (1, 2). It is characterized by burning mouth sensation or stomatodynia in patients with a clinically normal oral mucosa and without any particular disease (3, 4). BMS is also considered a type of neuropathic pain characterized by spontaneous, persistent, and recurrent symptoms. This burning pain can cause unpleasant feelings and emotional experiences, which are often positively correlated with the severity of BMS (5). Notably, individuals with chronic neuropathic pain are at risk of suicide, cigarette smoking and other mental health problems, significantly affects quality of life and places a socioeconomic and medical burden on patients and health care systems (6, 7). Various local, systemic and psychological factors have been found to be associated with BMS, but its etiology is not fully understood (4). Therefore, treatment of BMS has always been a challenging problem, and there is currently no recognized effective treatment method. The treatment primarily aims at eliminating the painful burning dysesthesia. The treatment methods mainly focuses on symptomatic treatment, including pharmacological management represented by clonazepam, nonpharmacological management represented by low-level laser therapy (LLLT), and psychological interventions such as cognitive behavioral therapy, and the efficacy is uncertain (1). Alpha-lipoic acid, topical clonazepam, gabapentin, and psychotherapy may provide modest relief of pain in BMS (8, 9). The side effects of pharmacological management are common because long-term administration usually required (10). Besides, psychotherapy usually requires psychologists to implement rather than dentists, and its professionalism and complexity limit its clinical application. Therefore, LLLT has become an important innovation in improving chronic pain of BMS in recent years due to its advantages of simple operation and almost no side effects.

Low-level laser therapy (LLLT) is also known as “cold laser” therapy or photobiomodulation therapy (PBMT). Using coherent laser light or light emitting diodes (LEDs) to generate red and near infrared light, which acts on targeted cells or tissues, producing analgesic, anti-inflammatory, and bio stimulatory effects (11). Compared with other forms of laser therapy used for ablation, cutting, and thermal coagulation of tissues, LLLT used light with lower energy density, hence it is referred to as “low-level” (12). Multiple studies have found the efficacy of LLLT in certain conditions, such as oral mucositis, recurrent herpes simplex infection, and BMS. When pharmacotherapy alone is ineffective, LLLT can serve as a complementary treatment option. The absence of any reported adverse effects is an advantage over conventional therapeutic modalities (13). A double-blind randomized controlled clinical trial found that burning sensation severity and quality of life in LLLT group were significant statistically better than placebo group after two LLLT sessions for 4 weeks (14). Another randomized controlled clinical trial included 78 BMS subjects found that LLLT reduced the symptoms of BMS patients who were randomly assigned to receive 9–10 LLLT sessions (15). A recent meta-analysis found that LLLT could reduce burning pain in patients with BMS, and have a positive influence on the quality of life and anxiety symptoms, without serious side effects, indicating that it may be an effective therapy for BMS (5).

In contrast, some scholars also believe that the effect of LLLT on BMS is only a placebo effect. A study found that the salivary levels of TNF-α and IL-6 in the LLLT group decreased significantly after 4 weeks of LLLT, but there was no significant difference in pain score (16). A randomized placebo controlled study included 23 BMS subjects who received 4 sessions for 2 weeks, and found that LLLT is as beneficial as placebo treatment in BMS patients, indicating a great emotional component of involvement in BMS symptomatology (17). To sum up, the effectiveness of LLLT in patients with BMS was still uncertain as the level of evidence-based findings was low and not comprehensive. More research is needed to verify whether LLLT has an analgesic effect on BMS, explore the influencing factors and mechanisms of its therapeutic efficacy.

Since psychological factors play an important role in the onset of BMS (18, 19), good immediate treatment effect can significantly improve patient satisfaction and alleviate anxiety, which is beneficial for further improving treatment effectiveness. It is worth noting that there was limited evidence in existing studies to evaluate the immediate efficacy of LLLT in treating BMS. This study focused on evaluating the immediate pain relief effect of LLLT in treating BMS, which will further perfect the research evidence of LLLT in the treatment of painful BMS.

The aim of this study is to evaluate the immediate analgesic effect of LLLT on BMS, and to explore the effects of age, gender, disease duration, pre-treatment pain level, primary/secondary, previous treatment, systemic diseases, smoking/alcohol, prosthetic restorations, xerostomia, and location of lesions factors on pain relief. The primary outcome of this study is overall efficiency. This study was reported following the STROBE guidelines as much as possible (20).

## 2 Materials and methods

### 2.1 Patient selection

A retrospective chart review of BMS patients who received treatment in the Dept. of Oral Medicine at the Hospital of Stomatology, Sun Yat-Sen University between May 2023 and February 2024, was conducted. The study protocol was approved by the Medical Ethics Committee of Hospital of Stomatology Sun Yat-Sen University (IRB AF/SC-07/v4.0). Due to the study was retrospective, we applied to the Medical Ethics Committee of Hospital of Stomatology Sun Yat-Sen University for exemption from the informed consent form of the subjects and obtained approval. The study has been registered on the chictr.org.cn website (ChiCTR2400091202).

Ninety-four BMS patients were included in this study. The inclusion criteria were as follows: (i)based on medical history and clinical examination, it can be diagnosed as BMS. the diagnostic criteria refer to International Classification of Headache Disorders 3rd edition (ICHD-3) (21): oral pain recurred for >2 h/days for >3 months, burning quality pain limited to superficial oral mucosa, oral mucosa is of normal appearance and clinical examination including sensory testing is normal, not better accounted for by another ICHD-3 diagnosis; (ii) visual analogue scale (VAS) is greater than or equal to 2; (iii) aged between 18 and 80 years old; (iv) has the ability to clearly judge and express the degree of pain; (v) be able to come to the hospital for regular follow-up visits as required and strictly follow medical advice; and (vi) no other oral mucous membrane diseases. The exclusion criteria were as follows: (i) pregnant or lactating women; (ii) accompanied by serious diseases of important organs such as heart, liver, kidney, and blood system; (iii) patients with a history of serious mental illness; or (iv) not cooperating with treatment or not following prescribed medication.

### 2.2 Low-level laser therapy

Painful BMS patients were offered LLLT laser treatment as an additional treatment (exposure time, 20 s; fluence, 2 J/cm^2^ per session; irradiance, 100 mW/cm^2^). A semiconductor laser was used as a light source (GYS-PDT-S2000, China Medical, 635 nm, 100 mW, continuous wave). The laser treatment was performed in a non-contact mode, with slow rotational movements over the painful areas.

### 2.3 Clinical evaluation of symptoms

Patient's pain level was assessed using the visual analogue scale (VAS) before and immediately after each LLLT session. The VAS is a line segment from 0 to 10, with two end points representing 0 (“no pain”) and 10 (“the most intense pain imaginable”). Patients were asked to place a mark on the line to rate their current level of pain. Pain reduction was calculated as the difference in VAS before and after LLLT. The pain response was designated a complete response (CR) if the pain reduction exceeds 90%, a significant response (SR) if a reduction exceeding 50% and less than 90%, a partial response (PR) if a reduction exceeding 25% and less than 50% and no response (NR) if a reduction less than 25% or an increase occurred. The overall efficiency rate was calculated using the following formula: (CR + SR + PR)/(CR + SR + PR + NR) × 100%. Patients' age, gender, disease duration, system diseases, history of prior treatment and sites of pain were retrieved from the electronic medical files.

### 2.4 Statistical analysis

Data were analyzed using SPSS version 20 (SPSS Inc., Chicago, IL). Quantitative data was statistically described using mean, median, standard deviation (SD). Classification data was statistically described using sample size and percentages. Paired-samples *t*-test was used to determine whether the pain reduction was significant. Multivariable binary logistic regression was used to analyze independent influencing factors of overall efficiency. A *P* value of 0.05 or less was considered significant.

## 3 Results

**Figure 1:**
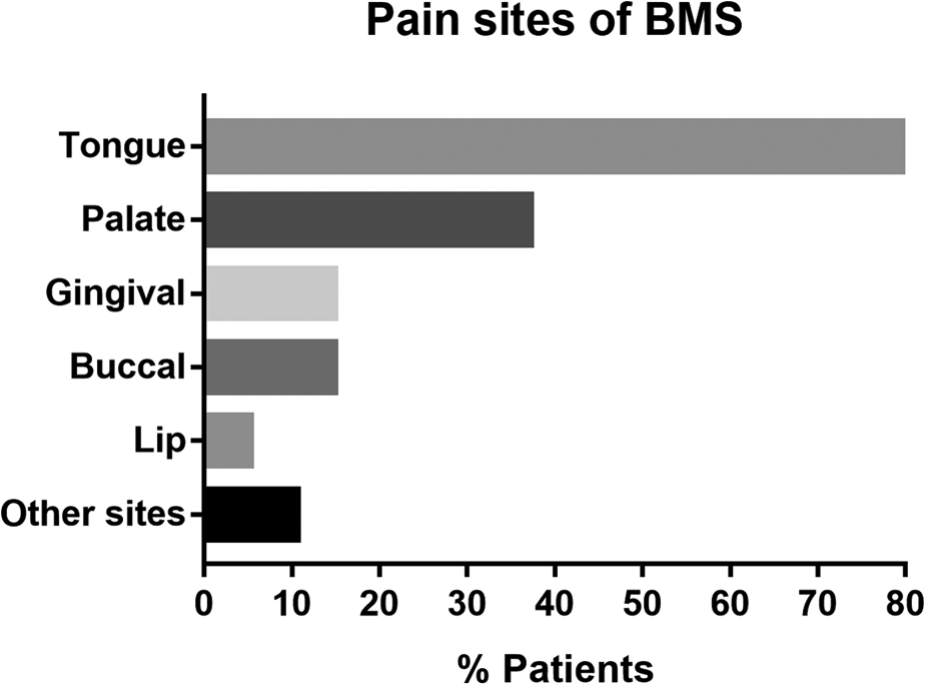
Pain sites of BMS. BMS, burning mouth syndrome. Other sites include pharynx, floor of the mouth, and pterygoid ligament area.

The baseline characteristics of the patients are presented in Table 1, Figure 1, Supplementary Table S1 and Figure S1. A total of 94 patients (twenty-three male and 71 females; age range 36–79, mean age 56.36 years) were included in the study. Among all 94 patients, 28 patients were accompanied by systemic diseases. The most common was heart disease (10/28) and hypertension (8/28), followed by thyroid disease (5/28) and lung disease (4/28), all of which were mild or well controlled. Regarding the history of prior treatment, 77.7% (73/94) of patients had received pharmacological therapy for BMS in our hospital or other hospitals, and were not satisfied with the treatment effect. Among the 74 patients who received pharmacological therapy, the majority (72/73) used a combination of multiple drugs, and 66 patients received treatment with three or more medications. A total of 11 drugs were used in this study, with mecobalamin having the highest usage rate among 70 patients, followed by 2.5% Sodium Bicarbonate Gargle Oryzanol (63/74), Oryzanol (57/74), and Recombinant Bovine Basic Fibroblast Growth Factor (rb-bFGF) (39/74), all of which had usage rates of higher than 50%. Among all 94 patients, 4 (4.3%) had a history of smoking or alcohol, 3 (3.2%) had metal or other prosthetic restorations, and 10 (10.6%) reported xerostomia as a concurrent complaint. The tongue and palate mucosa were the most common affected sites of BMS, and 79.8% of patients in this study had tongue lesions. Other sites included the pharynx, floor of the mouth, and pterygoid ligament area. About half of the BMS patients (46.8%) experienced pain in more than one intraoral site.

**Table 1:** Baseline characteristics of the patients (*n* = 94).

Characteristic	Statistical description
Age, years (mean ± SD, range)	56.36 ± 10.82, 36–79
Disease duration, months (mean ± SD, range)	11.95 ± 14.14, 0.2–60
VAS before treatment (mean ± SD, range)	5.30 ± 1.71, 2–10
Gender, *n*. (%)	
Female	71 (75.5)
Male	23 (24.5)
Systemic diseases, *n*. (%)	
No	66 (70.2)
Yes	28 (29.8)
History of prior treatment, *n*. (%)	
No	21 (22.3)
Yes	73 (77.7)
History of smoking or alcohol, *n*. (%)	
No	90 (95.7)
Yes	4 (4.3)
Metal or other prosthetic restorations, *n*. (%)	
No	91 (96.8)
Yes	3 (3.2)
Xerostomia, *n*. (%)	
No	84 (89.4)
Yes	10 (10.6)
Sites of pain *n*. (%)^a^	
Tongue	75 (79.8)
Palate	35 (37.2)
Gingival	14 (14.9)
Buccal	14 (14.9)
Lip	5 (5.3)
Other sites	10 (10.6)

^a^ Each patient may have more than one site of pain.

**Figure 2:**
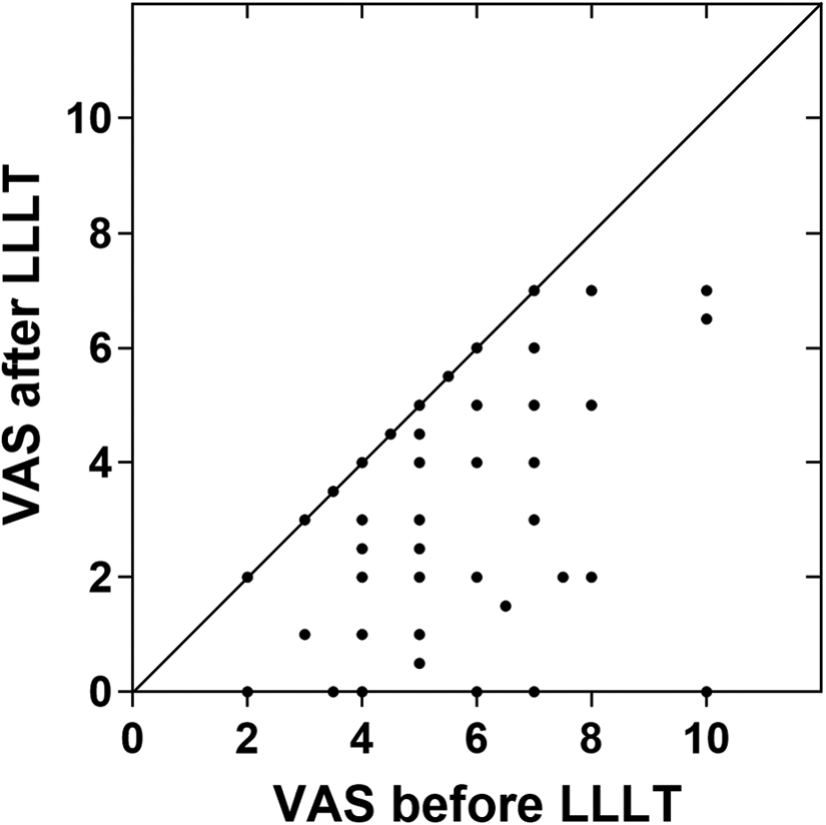
Self-assessment of BMS pain immediately before and after LLLT (*n* = 94). The data points located in the lower right triangle area represent pain reduction. The vast majority of patients experience pain relief after treatment and no cases of worsening pain have been detected. VAS, 0-to-10 numeric rating scale. LLLT, low-level laser therapy.

After standardized LLLT session, 71.3% (67 of 94) of patients reported an immediate pain decrease, 58.5% (55 of 94) patients achieved an effective response, with pain reduction over 25%. There were 39.4% (37 of 94) patients reported pain reduction of over 50% and 8.5% (8 of 94) patients reported complete pain relief. No occurrences of increased pain post-LLLT were documented (Figure 2). The primary outcome of this study is overall efficiency. The overall efficiency of patients in this study was 58.5% [95 CI%: 47.9%; 68.6%], indicating that LLLT has a statistically significant immediate analgesic effect on BMS patients.

**Figure 3:**
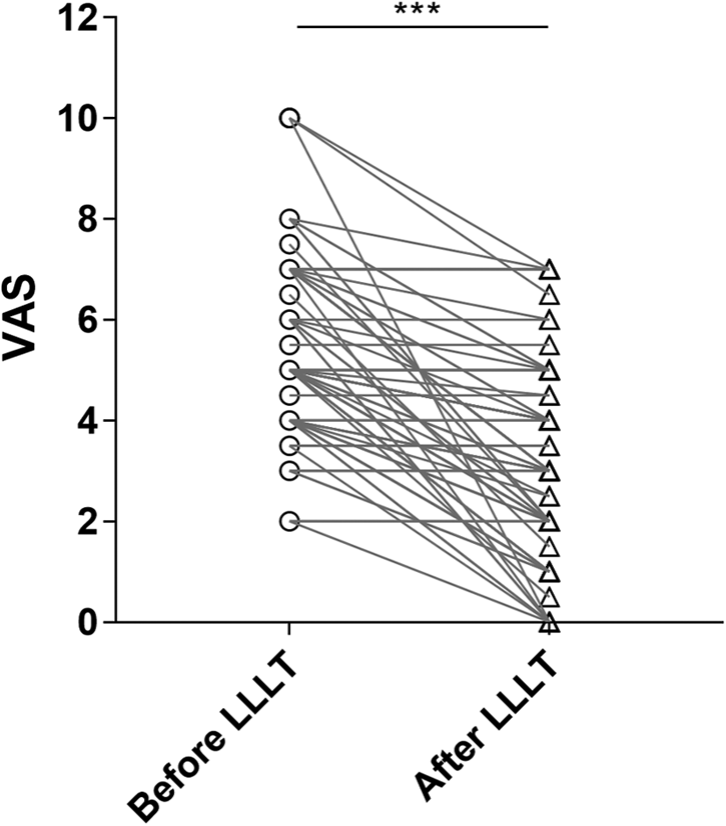
Pain reduction before and after LLLT. *p*-value: before LLLT vs. after LLLT was calculated by a paired-samples *t-*test. Note significant reduction in pain after LLLT. VAS, 0-to-10 numeric rating scale; LLLT, low-level laser therapy.

Compared to pre-LLLT treatment, the VAS immediately post-LLLT was significantly reduced (*P* < 0.001). Mean post-LLLT reduction in 0-to-10 pain VAS was 2.2 ± 2.0, indicating a post-LLLT reduction of 39.9% of the initial pain level (Figure 3).

**Figure 4:**
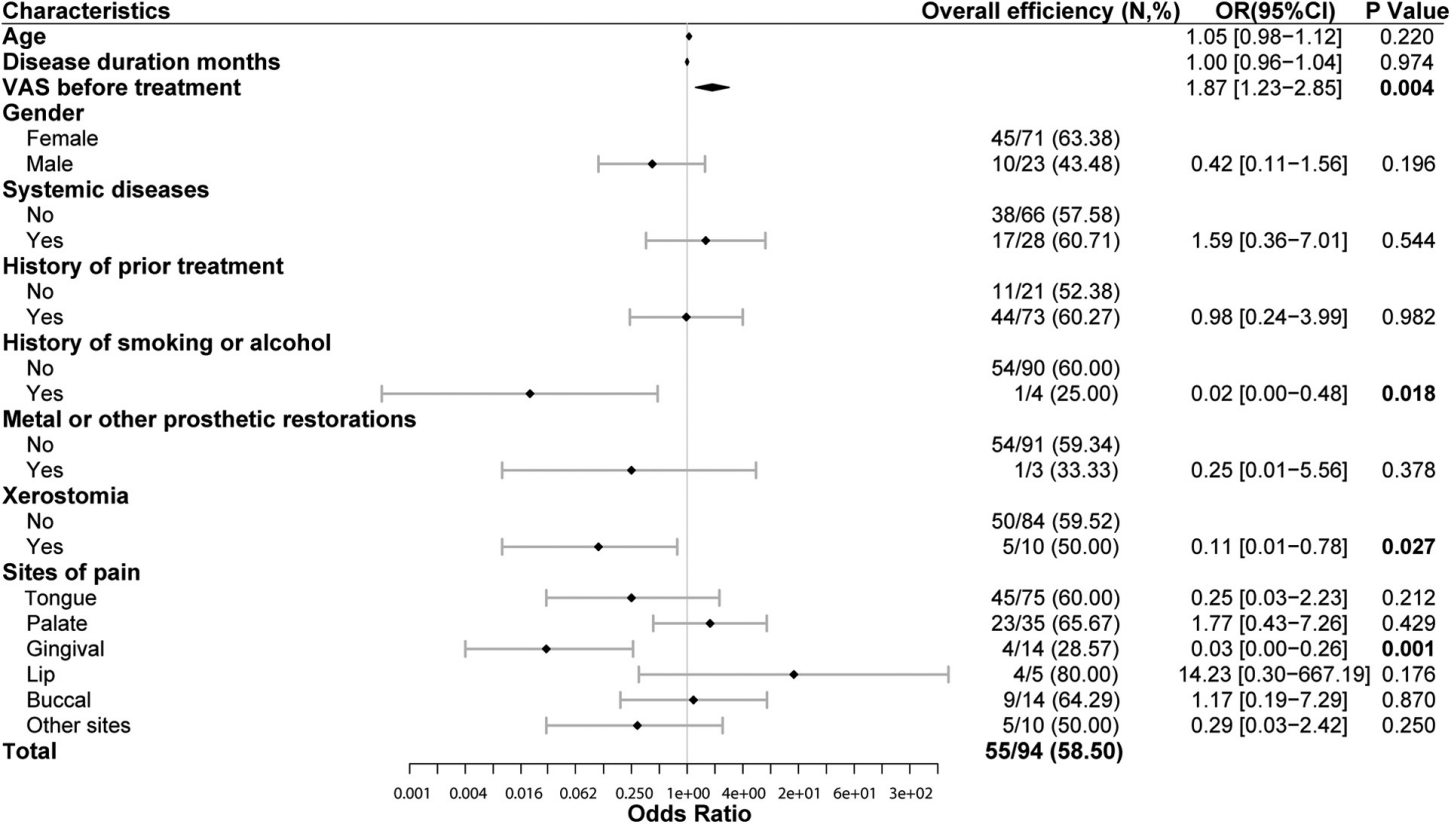
Overall efficiency of LLLT and its influencing factors (multivariable binary logistic regression). High VAS before treatment is closely related to significant efficiency of LLLT (OR = 1.87, *P* *=* 0.004). Patients with history of smoking or alcohol have lower overall efficiency of LLLT (OR = 0.02, *P* *=* 0.018). Patients with xerostomia have lower overall efficiency of LLLT than those without xerostomia (OR = 0.11, *P* *=* 0.027). The overall efficiency of patients with gingival lesions is lower (28.57%) than the average (58.5%) (OR = 0.03, *P* *=* 0.001). VAS, 0-to-10 numeric rating scale. LLLT, low-level laser therapy. OR, odds ratio. Overall efficiency = Number of people with pain relief over 25%/total number in this subgroup.

Our study observed the impact of 10 characteristics on overall efficiency, including age, gender, disease duration, system diseases, history of prior treatment, history of smoking or alcohol, metal or other aesthetic restorations, xerostomia, VAS before treatment and sites of pain. The multivariable binary logistic regression result showed that VAS before treatment, history of smoking or alcohol, xerostomia, gingival lesions were independent predictors of overall efficiency. Patients with higher VAS before treatment were more likely to benefit from LLLT treatment (OR = 1.87, *P* = 0.004). For every 1 score increased in VAS before treatment, the probability of overall efficiency increased by 0.87 folds. Patients with a history of smoking or alcohol (OR = 0.02, *P* = 0.018), as well as those with xerostomia (OR = 0.11, *P* = 0.027), have poorer response on LLLT treatment. The overall efficiency of gingival lesions is significantly lower (28.57%) than the average (58.5%) (OR = 0.03, *P* = 0.001), indicating that the overall efficiency of patients with gingival lesions is only 3% of that in patients of other lesions. As the most common site of BMS, the overall efficiency of tongue mucosa is not significantly different from other lesions (*P* = 0.21). Other characteristics (age, disease duration, gender, systemic diseases, history of prior treatment and metal or other aesthetic restorations) were not independent influencing factors on overall efficiency (Figure 4). The above findings can help us predicting treatment outcomes based on the basic characteristics of patients before treatment.

In all 94 LLLT sessions, no side effects or adverse reactions were noticed by the practitioner or reported by the patients.

## 4 Discussion

For The clinical features of patients in our survey shared many similarities with those reported previously. Usually, BMS has been described to be more prevalent in middle-aged and older women (50∼70y), with a female to male ratio varying from 3:1 to 16:1 (16). In our study, the female to male ratio was 3.1:1. Patients aged 50–70 accounted for 59.6% of the total number of patients. In previous studies, the probability of xerostomia in BMS patients was approximately 24.9∼30.2% (22, 23), which is higher than the percentage in this study (10.6%). This may be related to the humid subtropical monsoon climate and light dietary habits in Guangzhou. The tongue was affected in 79.8% of patients of our sample, in agreement with the literature which reports the tip and lateral borders of the tongue to be the sites most commonly affected by burning mouth (24, 25). The median pain VAS before LLLT was 5.30 (corresponding to a moderate intensity of symptoms) which is similar to the findings reported by Danhauer et al. (26).

After standardized LLLT session, 71.3% of patients reported an immediate pain decrease. The mean pain reduction was 2.2 ± 2.0 points on the 0-to-10 VAS (39.9% reduction from initial pain level). Compared to pre-LLLT treatment, the VAS immediately post-LLLT was significantly reduced (*P* < 0.001). The significant immediate pain relief effect greatly enhanced the patient's treatment confidence and relieved anxiety. Previous research has found that anxiety was one of the most common and frequently studied psychopathological disorders among BMS patients (27, 28). Therefore, relieving anxiety helps to achieve good treatment outcomes for BMS patients. A retrospective single-arm study (*n* = 30) recently published found that the immediate VAS of LLLT treatment decreased by an average of 75.3% (54.5%–92.2%) compared to before treatment. At the one week follow-up after treatment, the VAS slightly increased compared to immediately after treatment, but still significantly lower than the starting score (29). Another clinical trial (prospective, randomized, double-blind, placebo-controlled, *n* = 21) also treated BMS patients with LLLT and recorded the VAS before treatment, immediately after treatment, and 2 months after treatment. It was found that the VAS in the laser group decreased by 61.8% (before: 8.9; after: 5.5), while the placebo group decreased by 43.1% (before: 8.3; after: 5.5), indicating that the immediate pain relief effect of the laser group was better than that of the control group, and this advantage was further increased at the 2-month follow-up after treatment (laser group: 4.7, placebo group: 5.1) (30). In the above study, the immediate pain relief effect of LLLT on BMS patients was significantly better than that of the placebo group, and it still maintained a good analgesic effect during the follow-up period of 1 week to 2 months. We have reason to believe that the immediate pain relief effect of LLLT on BMS patients is not solely due to the placebo effect. On the other hand, BMS is already a disease heavily influenced by psychological factors, and good immediate pain relief effects can increase patients' treatment expectations, which in turn promotes disease healing. Of course, the duration of pain relief is also important, and we will collect this data in subsequent large-scale clinical studies to further refine our conclusions.

Here, we used the 635 nm laser because it is one of the most effective wavelengths for wound healing (31). Comparing the present results with other findings obtained in previous clinical studies on LLLT treatment of BMS, it was noted that the conclusions are consistent. Dos Santos et al. (32) evaluated the effect of LLLT (continuous wavelength, 660 nm, 0.8 J/point) on BMS. All the 10 patients recovered from the condition and there was a 58% decrease in pain severity. Arbabi-Kalati et al. (14) used 630 nm diode laser (power 30 mW) for 10 s twice a week. After 4 weeks of treatment, LLLT reduced burning sensation and improved the quality of life. Another similar study conducted LLLT at 660 nm (power 30 mW, irradiance 3 J/cm^2^) once a week for 4 weeks, and found that LLLT were effective therapies for relieving BMS symptoms (25). In existing studies, there is still a lack of standardized treatment plans for LLLT in treating BMS (5). Therefore, this study focuses on the efficacy evaluation of single LLLT treatment.

In this study, 77.7% of patients had a standardized prior treatment history and poor past treatment outcomes, which could be referred to as refractory BMS. It is noteworthy that LLLT still achieved quite good immediate analgesic effects in refractory BMS, with no statistically significant difference in efficacy compared to BMS patients without a history of previous treatment (60.3% vs. 52.4%, OR = 0.98, *P* = 0.982). This suggests that as a complementary option to traditional therapy, LLLT may have a novel mechanism of action. The mechanism by which LLLT influence BMS remain unclear. The gate theory, modulation of *β*-endorphin production, and anti-inflammatory effects are three most mainstream theories (33). An *in vitro* and *in vivo* study showed that laser irradiation was able to decrease ATP production, increasing the intracellular levels of ROS, inhibit the Na/K-ATPase and block the transmission of pain through sensory neurons to the brain (34). This is one of the possible mechanisms by which LLLT produces analgesic effects on BMS patients. Another research suggested that LLLT primarily modulates the endogenous opioids system to induced an analgesic effect on postoperative pain, and the reduction of IL-1β and TNF-α may play a role in the antinociceptive action of LLLT (35). A Male Wistar rats experiment found that He-Ne LLLT (632.8 nm, 2.5 J/cm^2^) inhibits the sensitization increase of nociceptors on the inflammatory process. The analgesic effect seems to involve hyperalgesia mediators (36). Further research is needed to provide a more detailed molecular mechanism of LLLT in treating BMS, which may help reveal the pathogenic mechanism of BMS.

In this study, we observed the impact of 10 individual characteristics of patients on overall efficiency and found that BMS patients with higher pain scores, without history of smoking or alcohol, without xerostomia, and without gingival lesions are more likely to benefit from LLLT treatment. In clinical practice, patients who meet the above criteria are more inclined to recommend LLLT treatment. Previous studies (5, 37) have mostly focused on the impact of factors such as total number of interventions, intervention frequency, laser parameters, and irradiation time on efficacy, with less attention paid to individual differences among patients. However, we believe that paying attention to individual differences between patients can help achieve personalized and precise treatment, and accurate treatment expectations can improve the quality of doctor-patient communication, which has important clinical value.

This study is a case-control study without a placebo control, which is the biggest limitation of this study. However, this study focused on immediate pain reduction. The interval between two pain assessments (immediately before vs. immediately after treatment) was only a few minutes. This paired control pre/post design served to partially offset the absence of a placebo control. In addition, 73 BMS patients in this study had received standardized pharmacologic treatment with poor efficacy, and no respond to previous treatments can also serve as another inherent control. Another limitation is the small number of patients. Nonetheless, despite the small sample size, a highly significant pain reduction was observed.

In conclusion, we are excited to find that the immediate pain relief effect of LLLT on BMS was significant, and its therapeutic effect was not affected by factors such as gender, age, disease duration, system diseases, history of prior treatment and metal or other aesthetic restorations. Meanwhile, BMS patients with higher pain scores, without history of smoking or alcohol, without xerostomia, and without gingival lesions are more likely to benefit from LLLT treatment. Moreover, due to the nonpharmacologic, patient-friendly, rapid pain relief, and almost no adverse reactions of LLLT, it has great value for promotion and application as a new treatment method for BMS treatment.

## Data Availability

The original contributions presented in the study are included in the article/Supplementary Material, further inquiries can be directed to the corresponding authors.
