# Correction: The immediate pain relief of low-level laser therapy for burning mouth syndrome: a retrospective study of 94 cases

**DOI:** 10.3389/froh.2025.1657781

**Published:** 2025-07-21

**Authors:** Wenxin Mu, Shanshan Li, Qian Lu, Juan Wang, Xiaoan Tao

**Affiliations:** ^1^ Hospital of Stomatology, Sun Yat-sen University, Guangzhou, China; ^2^ Guangdong Provincial Clinical Research Center of Oral Diseases, Sun Yat-sen University, Guangzhou, China; ^3^ Guanghua School of Stomatology, Sun Yat-sen University, Guangzhou, China

**Keywords:** low-level laser therapy, burning mouth syndrome, pain management, visual analogue scale, retrospective study

**Incorrect funding**

Grant number incorrect

An incorrect number was provided for [No. SL2023A04J01679)]. The correct number is [No. 2023A04J2164]. The original version of this article has been updated.

In the published article, there was a mistake in the Funding statement. The funding statement for the Guangzhou Municipal Science and Technology Bureau was displayed as “No. SL2023A04J01679”. The correct statement is “Guangzhou Municipal Science and Technology Bureau (No. 2023A04J2164)’’.

