# Supplementary figures and images for: The immediate pain relief of low-level laser therapy for burning mouth syndrome: a retrospective study of 94 cases
Source: Front Oral Health. 2024 Dec 18;5:1458329. doi: 10.3389/froh.2024.1458329 (PMC11688308; doi:10.3389/froh.2024.1458329)

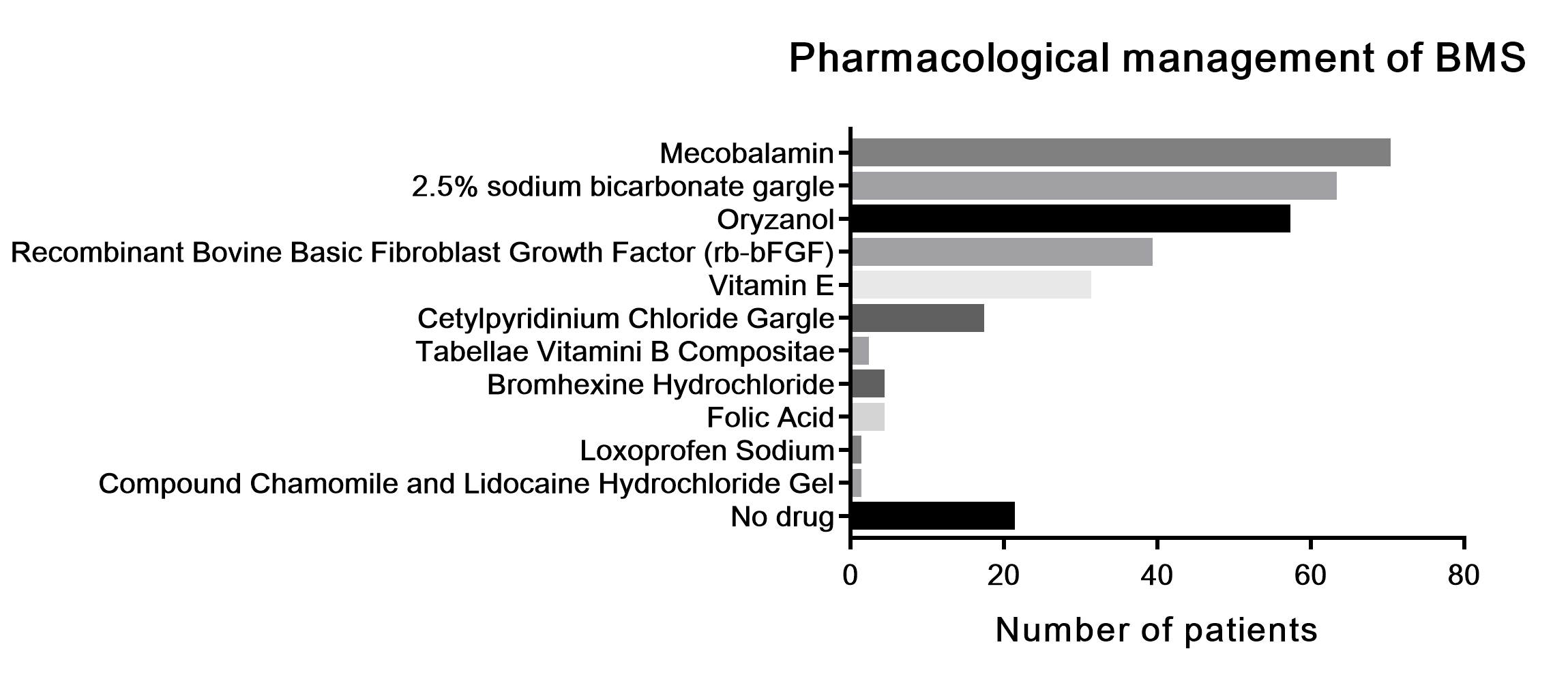

Supplement: Supplementary file 1 [file Image1.tif]
