# Supplementary material for: The immediate pain relief of low-level laser therapy for burning mouth syndrome: a retrospective study of 94 cases
Source: Front Oral Health. 2024 Dec 18;5:1458329. doi: 10.3389/froh.2024.1458329 (PMC11688308; doi:10.3389/froh.2024.1458329)
Supplement: Supplementary file 2 [file Table1.docx]

**Table S1.** Pharmacological management of the BMS patients

| **Medicines** | **Usage and dosage** |
| --- | --- |
| Mecobalamin | 0.5mg, PO, TID |
| 2.5% Sodium Bicarbonate Gargle | Gargle, TID |
| Oryzanol | 10mg, PO, TID |
| Rb-bFGF | External Application, TID |
| Vitamin E | 50mg, PO, TID |
| Cetylpyridinium Chloride Gargle | Gargle, TID |
| Tabellae Vitamini B Compositae | 1 tablet (containing 3mg vitamin B1, 1.5mg vitamin B2, 0.2mg vitamin B6), PO, TID |
| Bromhexine Hydrochloride | 8mg, PO, TID |
| Folic Acid | 5mg, PO, TID |
| Loxoprofen Sodium | 60mg, PO, TID |
| Compound Chamomile and Lidocaine Hydrochloride Gel | External Application, TID |

BMS, burning mouth syndrome. Rb-bFGF, Recombinant Bovine Basic Fibroblast Growth Factor.

PO, by mouth. TID, three times a day. QD, once a day.


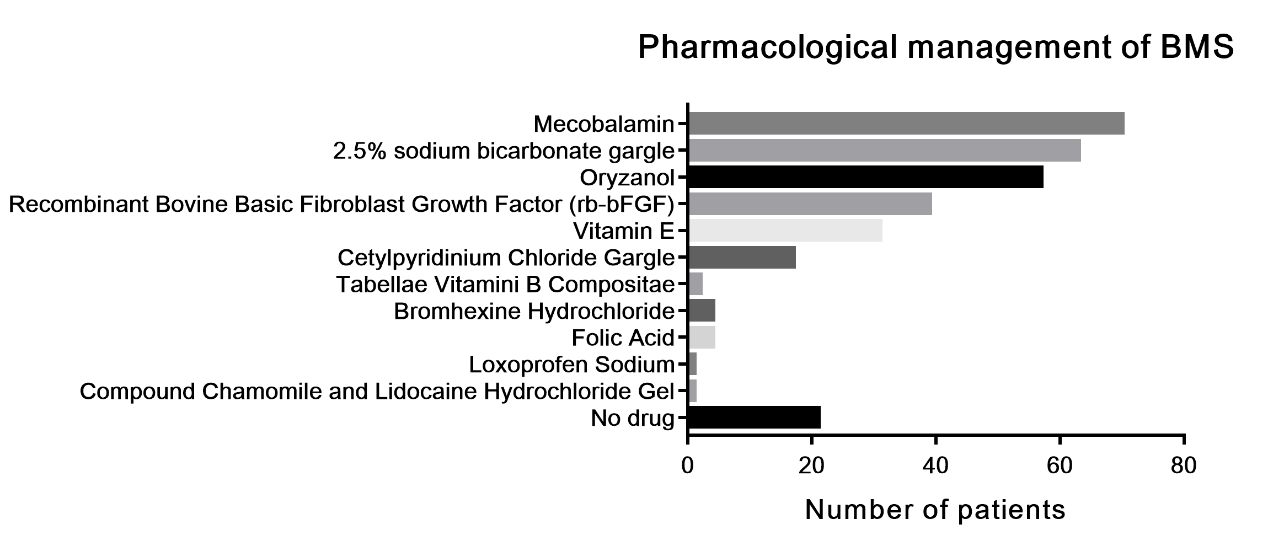


**Figure S1.** Pharmacological management of the BMS patients.

BMS, burning mouth syndrome.
